# Detecting scaphoid fractures in wrist injury: a clinical decision rule

**DOI:** 10.1007/s00402-020-03383-w

**Published:** 2020-03-03

**Authors:** Wouter H. Mallee, M. M. J. Walenkamp, M. A. M. Mulders, J. C. Goslings, N. W. L. Schep

**Affiliations:** 1 Department of Orthopaedic Surgery, Amsterdam University Medical Center, AMC, Meibergdreef 9, 1100 DD Amsterdam, The Netherlands; 2 Department of Trauma Surgery, Amsterdam University Medical Center, AMC, Amsterdam, The Netherlands; 3 Department of Trauma Surgery, Onze Lieve Vrouwe Gasthuis, Amsterdam, The Netherlands; 4 Department of Trauma and Hand Surgery, Maasstad Hospital, Rotterdam, The Netherlands; 5 Department of Orthopaedic Surgery, Amsterdam University Medical Center, AMC, Amsterdam, The Netherlands

**Keywords:** Scaphoid, Fracture, Diagnosis, Clinical evaluation, Decision rule, Predictors

## Abstract

**Introduction:**

The aim of this study was to develop and validate an easy to use clinical decision rule, applicable in the ED that limits the number of unnecessary cast immobilizations and diagnostic follow-up in suspected scaphoid injury, without increasing the risk of missing fractures.

**Methods:**

A prospective multicenter study was conducted that consisted of three components: (1) derivation of a clinical prediction model for detecting scaphoid fractures in adult patients following wrist trauma; (2) internal validation of the model; (3) design of a clinical decision rule. The predictors used were: sex, age, swelling of the anatomic snuffbox, tenderness in the anatomic snuffbox, scaphoid tubercle tenderness, painful ulnar deviation and painful axial thumb compression. The outcome measure was the presence of a scaphoid fracture, diagnosed on either initial radiographs or during re-evaluation after 1–2 weeks or on additional imaging (radiographs/MRI/CT). After multivariate logistic regression analysis and bootstrapping, the regression coefficient for each significant predictor was calculated. The effect of the rule was determined by calculating the number of missed scaphoid fractures and reduction of suspected fractures that required a cast.

**Results:**

A consecutive series of 893 patients with acute wrist injury was included. Sixty-eight patients (7.6%) were diagnosed with a scaphoid fracture. The final prediction rule incorporated sex, swelling of the anatomic snuffbox, tenderness in the anatomic snuffbox, painful ulnar deviation and painful axial thumb compression. Internal validation of the prediction rule showed a sensitivity of 97% and a specificity of 20%. Using this rule, a 15% reduction in unnecessary immobilization and imaging could be achieved with a 50% decreased risk of missing a fracture compared with current clinical practice.

**Conclusions:**

This dataset provided a simple clinical decision rule for scaphoid fractures following acute wrist injury that limits unnecessary immobilization and imaging with a decreased risk of missing a fracture compared to current clinical practice.

**Clinical prediction rule:**

1/(1 + EXP (−(0.649662618 × if man) + (0.51353467826 × if swelling anatomic snuffbox) + (−0.79038263985 × if painful palpation anatomic snuffbox) + (0.57681198857 × if painful ulnar deviation) + (0.66499549728 × if painful thumb compression)−1.685).

**Trial registration:**

Trial register NTR 2544, www.trialregister.nl.

**Electronic supplementary material:**

The online version of this article (10.1007/s00402-020-03383-w) contains supplementary material, which is available to authorized users.

## Introduction

Scaphoid fractures are difficult to diagnose, especially in the acute setting. Due to the risk of complications, such as non-union and radiocarpal arthritis, patients with clinically suspected scaphoid fractures with normal radiographs are initially immobilized with a splint until further diagnostics are performed. On average, 80% of these patients do not have a scaphoid fracture [8], resulting in substantial overtreatment (e.g., immobilization), unnecessary follow-up imaging and substantial impact on work. Clinical assessment of possible scaphoid fractures in the emergency department (ED) is limited due to a lack of evidence supporting adequate tests [7].

Improving the clinical selection of patients that require imaging and immobilization is warranted and a well-designed clinical decision rule could be the solution. Combining several clinical tests such as tenderness in the anatomic snuffbox and painful longitudinal thumb compression tests has already proven to increase the diagnostic accuracy of the clinical assessment of scaphoid injury [3, 10, 11]. However, these studies were either underpowered, of uncertain methodology or were impractical for implementation in daily practice.

The aim of this study was to develop and validate an easy to use clinical decision rule, applicable in the ED that limits the number of unnecessary splinting and diagnostic follow-up in suspected scaphoid injury, without increasing the risk of missing a fracture.

## Methods

### Study design

This study was part of a comprehensive research project, the Amsterdam Wrist Rules. This study included all wrist injuries to identify predictors for a distal radius or a scaphoid fracture [20]. A prospective multicenter study was performed, consisting of three components: (1) derivation of a clinical prediction model for detecting scaphoid fractures in patients following wrist trauma; (2) internal validation via bootstrapping and (3) design of a clinical decision rule. The study was conducted at the emergency departments of five Dutch hospitals from November 11, 2010 to June 25, 2014. The participating hospitals included one academic hospital and four regional teaching hospitals. Our Institutional Review Board approved this study without the need for informed consent. The trial was registered at the Dutch Trial Registration prior to start of inclusion (NTR 2544, www.trialregister.nl).

**Fig. 1:**
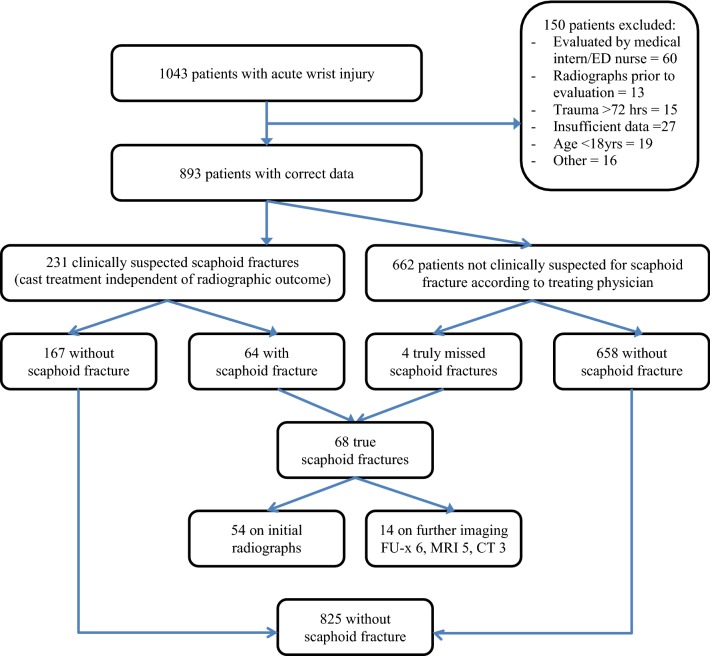
Flowchart of patient inclusion and exclusion

The entire dataset of the Amsterdam Wrist Rules comprised a consecutive series of all adult patients (≥ 18 years) presenting to the emergency department (ED) with acute wrist injury (within 3 days after the initial trauma). For this trial, all patients that were suspected for a scaphoid fracture according to the treating (ED) physician were included. In addition, the entire Amsterdam Wrist Rules dataset was searched for possible missed scaphoid fractures with a time frame of at least 3 months after initial ED presentation. All patients had radiographs (one posteroanterior, one true lateral and, if suspected of a scaphoid fracture, one semipronated oblique and posteroanterior view of the wrist in ulnar deviation). Patients were excluded if radiographs were performed prior to clinical evaluation; if any previous initial treatment was started in another hospital; if evaluation was performed by a nurse or general practitioner/house doctor; in case of multi-trauma or severe pain preventing examination; and any cognitive disorders limiting accurate response to questions. All patients with a true scaphoid fracture (both initially visible and occult) and all patients without a scaphoid fracture were divided into two groups for comparison of characteristics (Fig. 1: flowchart).

ED physicians or residents in (orthopedic) trauma clinically evaluated all patients by using a specially designed case record form (CRF) prior to initial radiographs. Since knowledge of anatomy and tests was essential, education was given by presentations and laminated descriptive sheets to the residents and ED physicians prior to the study initiation. Both demographics and clinical tests were implemented in the CRF (Table 1). These variables were based on a recent review [7]: age, sex, presence of swelling in the anatomic snuffbox (ASB), tenderness in the ASB, painful longitudinal thumb compression, scaphoid tubercle tenderness and pain over scaphoid with ulnar deviation. Definitive management was not interfered by the outcome of the CRF. An immobilizing cast or splint was given to those patients who were clinically suspected by discretion of the ED physician. The data on the CRFs were extracted by two researchers.

**Table 1:** Predictors and tests

Predictor	Outcome
Sex	Y/N
Age (per year)	Continuous
Swelling in ASB	Y/N
Tenderness in ASB	Y/N
Painful scaphoid with longitudinal thumb Compression	Y/N
Scaphoid tubercle tenderness	Y/N
Pain with ulnar deviation	Y/N

The primary outcome measure was the presence of a scaphoid fracture, diagnosed on initial radiographs, during re-evaluation after 1–2 weeks with repeated clinical evaluation (painless anatomical snuffbox and no pain with longitudinal thumb compression) and/or repeated radiographs or on additional imaging (MRI or CT). A fissure and an avulsion were classified as a fracture. The attending orthopedic trauma surgeon and/or a resident in orthopedic or trauma surgery and a radiologist evaluated the images during the trauma meeting the following day, they received normal clinical information and were blinded to the content of the case record forms.

### Sample size and statistical analysis

A common rule of thumb to determine the sample size of the development of a prediction model is ten events (true scaphoid fractures) per variable [17]. With seven variables, the inclusion of 70 patients with a true scaphoid fracture was required.

For efficient statistical analysis [2, 5, 14], we used imputation techniques to impute the missing values (*aregImpute* function from the *Hmisc* library, R, version 3.0.1). For each variable containing missings, the *aregImpute* package draws values from a random sample from the non-missing values with replacement. Using this data, aregImpute fits a flexible model that predicts the missing target variable while finding its optimum transformation. Each missing variable is then imputed with the observed value whose predicted transformed value is closest to the predicted transformed value of the missing variable. We considered an imputation model that included all dichotomous variables. The set of first imputations was used for the analyses.

Descriptive statistics were performed to summarize the baseline characteristics. Categorical variables were presented as frequencies and percentages. Normality of the data was assessed by visually inspecting the normality plots. Parametric data were presented as mean and standard deviation (SD) and non-parametric data were presented as median and interquartile range [IQR]. Differences in patient characteristics between the groups with and without a scaphoid fracture were calculated using the Mann–Whitney *U* test for continuous data and the Chi-square test for categorical data. For each predictor, the sensitivity and specificity were calculated according to standard formulas.

### Model development and internal validation

We derived a clinical prediction model for scaphoid fractures using data on all patients with a clinically suspected fracture including missed fractures, enrolled in the study. A multivariate logistic regression model with all seven potential predictors was fit. This full model was reduced using a stepwise backward elimination process based on a liberal *p* value of 0.15 [15]. The coefficient determines the effect of that predictor on the probability of a true scaphoid fracture. The coefficient of each variable represents the amount of change in the probability of a scaphoid fracture. A positive coefficient increases and a negative coefficient decreases the probability of a fracture. To estimate the internal validation of performance, we used bootstrapping (500 replications). Bootstrapping was used to quantify the optimism of the prediction model by mimicking the process of sampling from the underlying population: the difference between performance in the bootstrap sample and performance in the original sample. A shrinkage factor, also obtained by bootstrap validation, was used for multiplication of the regression coefficients [16, 18].

To estimate the ability of the model to discriminate between patients with and without a fracture, we calculated the areas under the receiver operating characteristics curve (AUC). The AUC ranges from 0.5 to 1, with a higher score indicating more accurate predictions. The model was also evaluated for their agreement between predicted fractures and observed fractures. This is otherwise known as the model calibration and was assessed by plotting the predicted probability of a fracture and the observed frequency of fractures. The ideal slope of such a plot is 1, indicating perfect agreement between observed and predicted risks [15].

### Clinical decision rule

A clinical prediction *model* provides an estimated risk of a certain outcome. A clinical decision *rule* goes one step further and links a recommendation to the predicted risk. In this study, the recommendation would be to request a radiograph yes or no. If yes, immobilization with a cast/splint is inevitable. A clinical decision rule therefore requires a cutoff value for the predicted probability of a fracture to classify patients as low or high risk (or recommend radiograph yes or no). We decided beforehand to select a cutoff value at which the sensitivity of the rule would not drop below 95% to minimize the risk of missing fractures.

To assess the effect of the rule, the number of overtreatment and undertreatment was calculated by applying the rule to the patients that were presumed clinically suspected for a scaphoid fracture by discretion of the ED physician (current clinical practice). The number of missed fractures and the number of overtreatment after the rule was applied were compared to current clinical practice.

## Results

### Patient characteristics

A total of 1043 adult patients with acute wrist injury were included in this study; 893 patients were eventually eligible for analysis. Sixty-eight patients (7.6%) were diagnosed with a scaphoid fracture, 54 patients (79%) during initial presentation and 14 patients (21%) during follow-up (radiographs 6, MRI 5, CT 3) (Fig. 1). Patients with a scaphoid fracture were significantly younger (*p* = 0.001) and males were overrepresented (*p* < 0.001), compared to patients without a scaphoid fracture. For patients characteristics see Table 2. A fall on the outstretched hand (FOOSH) was the trauma mechanism in 66% of the fractures.

**Table 2:** Patient characteristics

	All patients (*N* = 893)	Scaphoid fractures (*N* = 68)	Non scaphoid fractures (*N* = 825)	*P* value fracture vs non fracture
Median age (IQR)	50 (31–63)	35 (23.1–58.5)	50 (32.6–63.7)	0.001
Male	40%	62%	38.2%	< 0.001
Trauma to dominant side	48%	45.9%	48.2%	0.731

*IQR* interquartile range

At the discretion of the physician, 231 patients were clinically suspected of a scaphoid fracture, and this group included 64 scaphoid fractures. Four scaphoid fractures (5.9%) were missed and did not receive initial immobilization due to lack of clinical signs (e.g., no anatomical snuffbox tenderness and no pain during axial compression of the thumb). These patients were discharged with pressure bandage and presented themselves within 1–4 weeks after ED presentation with persistent wrist pain. The missed fractures were three distal scaphoid fractures (Herbert type A1-2) that received cast immobilization and one complete waist fracture (Herbert type B2) that did not unite and received open reduction and screw fixation after 4 months.

There were no missing values for age, sex and tenderness in the ASB. For swelling of the ASB (4 missing), scaphoid tubercle tenderness (6 missing), painful ulnar deviation (1 missing) and painful longitudinal thumb compression (6 missing), missing data were imputed accordingly.

### Results of individual tests

Table 3 shows the diagnostic accuracy, coefficients and odds ratios for each individual predictor. The diagnostic accuracy of tenderness in the ASB showed a sensitivity of 0.71 and a specificity of 0.25. For longitudinal compression of the thumb, sensitivity and specificity were 0.92 and 0.56, respectively. This test had the biggest effect on the clinical prediction rule, since its coefficient was the highest (0.8544). Pain with ulnar deviation resulted in a sensitivity of 0.82 and specificity of 0.34.

**Table 3:** Individual test results

Predictors	Sensitivity	Specificity	Coefficients (95% CIs)	*P* values	Odds ratios (95% Cis)
Age	n a	n a	0.0035 (−0.01296–0.01996)	0.6793	n a
Sex^a^	n a	n a	0.8347 (0.2306–1.4388)	0.0068	2.3041 (1.26–4.22)
Swelling ASB^a^	0.5224	0.6707	0.6598 (0.0063–1.3133)	0.0478	1.9345 (1.01–3.72)
Tenderness in ASB^a^	0.7059	0.2455	−1.0155 (−1.8360; −0.1950)	0.0153	0.362 (0.16–0.82)
Scaphoid tubercle Tenderness	0.6818	0.5524	0.1333 (−0.6423–0.9089)	0.7361	n a
Painful ulnar deviation^a^	0.8235	0.3373	0.7411 (0.0083–1.4739)	0.0475	2.0983 (1.01–4.37)
Painful longitudinal thumb compression^a^	0.9242	0.5583	0.8544 (0.0786–1.6302)	0.0309	2.35 (1.08–5.1)

*n a* = not applicable, *ASB* = anatomic snuffbox

^a^Included in the final rule

### Model development

The final clinical decision rule included five variables: sex, swelling of the ASB, tenderness of the ASB, painful ulnar deviation and painful longitudinal compression of the thumb (Table 3). The area under the curve (AUC) was 0.72 (95% CI 0.65–0.78), after correcting for optimism by bootstrapping. The calibration of the model was 1 (95% CI 0.59–1.40), indicating perfect agreement between predicted and observed fractures. The final formula for calculating the predicted chance of a true scaphoid fracture is shown in Table 4.

**Table 4:** Clinical prediction rule for detecting true scaphoid fractures

Linear Predictor	(0.649662618 × if man) + (0.51353467826 × if swelling anatomic snuffbox) + (−0.79038263985 × if painful palpation anatomic snuffbox) + (0.57681198857 × if painful ulnar deviation) + (0.66499549728 × if painful thumb compression) −1.685
Clinical prediction formula	1/(1 + EXP (− linear predictor))

### Outcome of the decision rule

If a threshold of 15% (the probability of a fracture is ≥ 15%) was applied, 66 scaphoid fractures would have been identified correctly, 2 scaphoid fractures would have been missed (1 minor avulsion of the tubercle (type A1) and 1 incomplete waist fracture (type A2) in anatomic position) and 36 patients would not have had further imaging and immobilization (Table 5). This results in a 15% reduction of further imaging and a 50% reduction of missed fractures. The sensitivity and specificity were 97% and 20%, respectively; positive and negative predictive values were 0.33 and 0.94, respectively; the prevalence of true fractures among clinically suspected fractures increased from 0.27 to 0.33.

**Table 5:** 2 × 2 table of the clinical prediction rule

	True fracture	No fracture	Total
Rule +	66	133	199
Rule −	2	34	36
Total	68	167	235
Positive predictive value	66/199 = 0.33		
Negative predictive value	34/36 = 0.94		
Sensitivity	66/68 = 0.97		
Specificity	34/167 = 0.20		
Prevalence without rule: 0.27 (64/235)	Prevalence with rule: 0.33 (66/199)		

## Limitations

First, the derived scaphoid rule is highly sensitive (97%), but the specificity is only 20%. Therefore, overtreatment will still be an issue in clinically suspected scaphoid fractures. The lack of specificity is frequently addressed in scaphoid fracture diagnostics [3, 7]; however, the results of this study show a reduction in unnecessary diagnostics and treatment of more than 15%.

Second, the AUC of this clinical prediction model after internal validation rule is 0.72. This means that the predicted probability of the rule showed a fair discrimination between patients with and without a scaphoid fracture. The higher the AUC, the better is the discriminative value of the rule. Bootstrapping methods provide bias-corrected estimates of the performance of a clinical prediction model and are recommended for internal validation [1]. However, external validation was not performed. Validation is most reliable when it is performed in a different patient population, and therefore prior to implementation of this clinical decision rule, it is necessary to externally validate this rule in a different patient population in other hospitals [1, 9].

Third, 70 scaphoid fractures were required based on the ‘rule of thumb’ in determining the sample size of a clinical prediction rule development (10 events per variable). This study included 68 patients with a proven scaphoid fracture. However, we believe two additional events would not have changed the outcome of the analysis.

The majority of the included patients did not undergo further imaging besides the standard radiographs. Therefore, it is a possibility that other occult fractures besides the scaphoid were missed. The interval between ED presentation and data collection should detect most patients who had persistent pain after discharge and minimized the risk of missed fractures.

A continuing issue in scaphoid research is the lack of an adequate reference standard to detect a true fracture. This study used different standards, MRI/CT/follow-up radiographs, based on the local preferences of the hospital. It is known that none of the modalities are 100% accurate [8, 11] and, therefore, misdiagnosis is still possible. Ultimately, all patients with a clinically suspected scaphoid fracture should receive either CT or MRI to obtain definitive diagnosis.

Using subjective measures such as physical examination introduces a possible lack of interobserver agreement. This trial did not incorporate an interobserver reliability study. Prior to the study, informative presentations and laminated descriptive sheets were provided concerning the anatomy and tests in clinical evaluation to limit inadequate application of the rule as much as possible.

## Discussion

With this study, we developed a highly sensitive clinical decision rule that is able to select those patients presenting at the ED with wrist trauma that require further imaging and treatment for a suspected scaphoid fracture. Moreover, when applying the Amsterdam Scaphoid Rule, a reduction of 15% in radiographs and unnecessary immobilization is possible, while reducing the number of missed fractures with 50%.

Similar to the Amsterdam Wrist Rules, this scaphoid clinical decision rule will be incorporated in the same smartphone application to simplify its use. The use of a mobile application has recently been studied in applying the Ottawa Ankle Rules [12]. It proved to increase its adherence significantly, which results in improving documentation of key clinical data. Using a mobile application also tackles the known barrier of forgetting details of the decision rule [19].

In order to link a recommendation to the derived prediction model, it is necessary to set a threshold value. The key is to find a reasonable balance between reduction of unnecessary overtreatment and imaging, and the risk of missing fractures. Several thresholds were used to determine the most suitable in this situation with the goal to keeping sensitivity above 95%. With a sensitivity of 97%, this study showed that with a threshold of 15% fracture probability, only 2 of 68 fractures were missed compared to 4 of 68 scaphoid fractures with current clinical practice. Thus, this scaphoid rule reduces the number of missed fractures with 50% and reduces unnecessary follow-up with 15%.

This is a large prospective study on the diagnosis of wrist injuries including 68 scaphoid fractures. The variables for clinical assessment were selected by performing a thorough systematic review [7] and analysis encompasses the use of robust statistical and study design methodology [15–17]. The inclusion of all acute wrist injuries ensured that all scaphoid fractures were included in the analysis to get an accurate representation of clinical practice. The statistical models have been thoroughly tested, for this study as well as previously for the Amsterdam Wrist Rules [13, 20].

Several findings from previous literature, like the fact that scaphoid fractures mainly occur in young male patients, are emphasized in this study [3, 4, 6]. However, controversial results were also detected, especially on the diagnostic performance characteristics of individual tests. A tender anatomic snuffbox and painful longitudinal thumb compression have been described as being highly sensitive for detecting scaphoid fractures [3, 10]. Duckworth et al. showed a 100% sensitivity of ASB tenderness [3]. In contrast, in this study we found a sensitivity of 71% for ASB tenderness. Moreover, ASB tenderness was not present in 20 of 68 (29.4%) scaphoid fractures. In addition, the number of occult fractures was remarkably higher in our data (21% vs 11%). An explanation for the latter is that the current study initially included all wrist injuries; this ensured the inclusion of four scaphoid fractures that were missed with current clinical assessment. Previous studies included patients that were ‘clinically suspected for a scaphoid fracture’, meaning that there was already a selection prior to inclusion. These selection methods were not described and thus induce selection bias.

Clinical assessment of patients with wrist injury can be protocolized with this decision rule. Moreover, with the Amsterdam Scaphoid Rule the risk of missing a fracture is lower than in current clinical practice. Therefore, it has the potential to reduce unnecessary immobilization and diagnostic follow-up without increasing the risk of missed fracture. It can thus provide physicians at the ED an easy and effective tool to select patients with suspected scaphoid fractures for radiography. If the rule does not suggest radiographical evaluation and immobilization, we suggest patients are either treated with a supportive bandage/tubi grip or discharged without treatment and to report back to the outpatient clinic when symptoms persist after 2 weeks. External validation and implementation of this rule will be subject of further research.

## Electronic supplementary material

Below is the link to the electronic supplementary material.

Supplementary file1 (PPTX 248 kb)
