# Supplementary material for: Detecting scaphoid fractures in wrist injury: a clinical decision rule
Source: Arch Orthop Trauma Surg. 2020 Mar 3;140(4):575–81. doi: 10.1007/s00402-020-03383-w (PMC7109163; doi:10.1007/s00402-020-03383-w)
Supplement: Supplementary file 1 — Supplementary file1 (PPTX 248 kb) [file 402_2020_3383_MOESM1_ESM.pptx]

## Slide 1
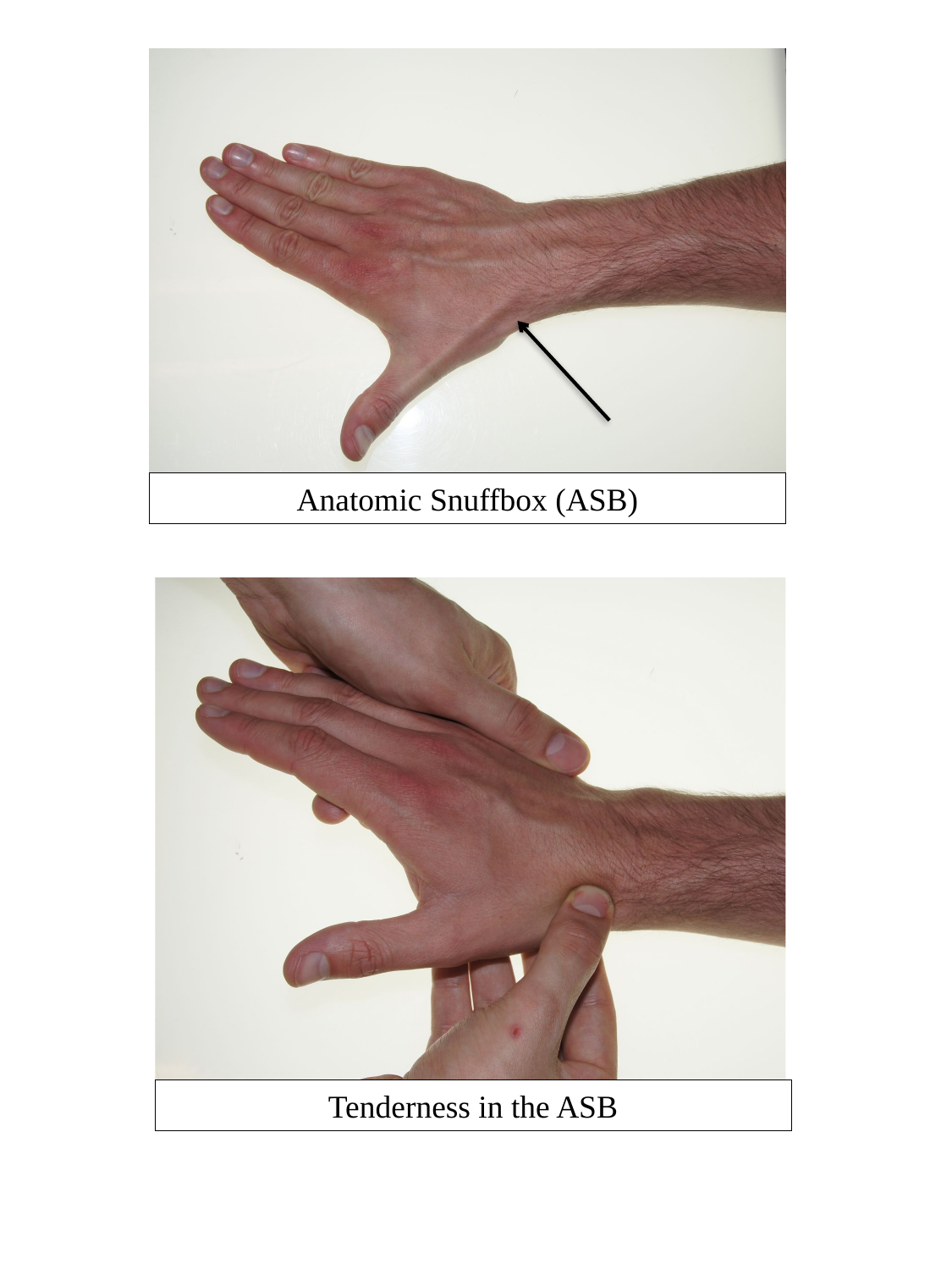

Anatomic Snuffbox (ASB)
Tenderness in the ASB

## Slide 2
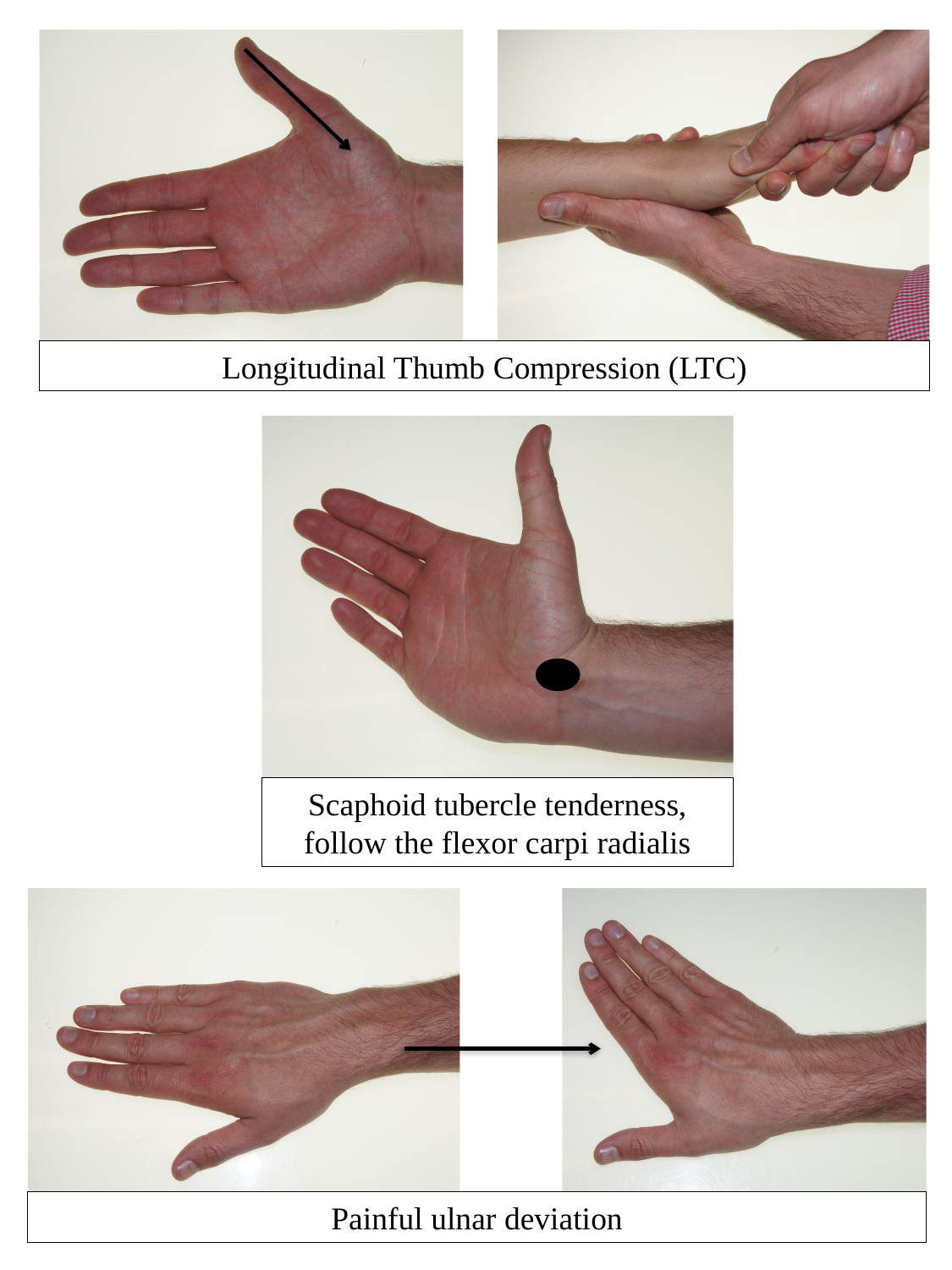

Longitudinal Thumb Compression (LTC)
Scaphoid tubercle tenderness, follow the flexor carpi radialis
Painful ulnar deviation
